# Exploring the support needs of Australian parents of young children with Usher syndrome: a qualitative thematic analysis

**DOI:** 10.1186/s13023-024-03125-w

**Published:** 2024-03-21

**Authors:** L. Johansen, F. O’Hare, E. R. Shepard, L. N. Ayton, L. J. Pelentsov, L. S. Kearns, K. L. Galvin

**Affiliations:** 1 UsherKids Australia, Mordialloc, VIC Australia; 2 Department of Optometry and Vision Sciences, The University of Melbourne, Parkville, VIC Australia; 3 Department of Surgery (Ophthalmology), The University of Melbourne, Parkville, VIC Australia; 4 Centre for Eye Research Australia, East Melbourne, VIC Australia; 5 Clinical and Health Sciences, University of South Australia, Adelaide, SA Australia; 6 Department of Audiology and Speech Pathology, Faculty of Medicine, Dentistry and Health Sciences, The University of Melbourne, Parkville, VIC Australia; 7 Royal Victorian Eye and Ear Hospital, East Melbourne, VIC Australia

**Keywords:** Usher syndrome, Parents, Support needs, Interview study, Rare disease, Disease burden

## Abstract

**Background:**

Advancements in genetic testing have led to Usher syndrome now being diagnosed at a much earlier age than in the past, enabling the provision of early intervention and support to children and families. Despite these developments, anecdotal reports suggest there are substantial gaps in the services and supports provided to parents of children with Usher syndrome. The current study investigated the support needs of parents of children with Usher syndrome Type 1 when their child was aged 0 to 5 years.

**Method:**

Purposive sampling was used, and six semi-structured interviews were conducted with Australian parents of children with Usher syndrome, Type 1. Data was analysed using modified reflexive thematic analysis.

**Results:**

Four key themes were identified as being central to the support needs of parents of children with Usher syndrome aged 0 to 5 years. (1) Social Needs referred to parents’ need for various sources of social support, (2) Informational Needs described the lack of information parents received regarding Usher syndrome from treating professionals, (3) Practical Needs included supports needed to assist parents in managing the day-to-day tasks of caring for a child with a disability, and (4) Emotional Needs represented the emotional support (both formal and informal) that parents needed to be a positive support to their child.

**Conclusions:**

Findings provide rich information for relevant support groups, policy makers, individual healthcare professionals, and professional governing bodies regarding the education of stakeholders and the development and implementation of best-practice treatment guidelines.

## Background

Usher syndrome is an autosomal recessive genetic condition [34] that affects approximately 1 in 6000 individuals globally [18]. Although rare, it is the leading cause of deafblindness worldwide [17]. The syndrome is characterised by partial-to-total hearing loss due to abnormal development of cochlear hair cells, and progressive vision loss due to degenerative changes of the retina involving the photoreceptor cells (referred to broadly as retinitis pigmentosa). Some individuals experience vestibular dysfunction due to abnormal development of vestibular hair cells, which results in difficulties with balance, coordination, and gross motor development [34]. Historically, the diagnosis of Usher syndrome was made on the clinical presentation of retinal changes and/or vision impairment in a patient who was already diagnosed with a hearing impairment. With recent advances in genetic testing technologies, children are now being diagnosed with Usher syndrome in the first years of life, before any signs of vision loss are evident [10].

Three clinically recognized subtypes of Usher syndrome exist, with a fourth atypical type recently proposed [33]. The types vary in terms of the age of onset, severity, rate of disease progression, and presence of vestibular dysfunction [23]. Type 1 is the most severe, due to the presence of profound congenital deafness, childhood onset of retinitis pigmentosa, and vestibular dysfunction. Types 2 and 3 are characterised by less severe congenital sensorineural hearing loss and a lower likelihood of vestibular dysfunction. These types are also characterised by the later onset of retinitis pigmentosa, with progressive vision loss typically beginning in the second decade of life for Type 2 and the fourth decade for Type 3. Type 4 has only recently been identified through molecular analysis and is associated with middle-to-late adult onset of progressive hearing and vision loss [19, 33].

There is currently no cure for Usher syndrome. The hearing impairment is typically managed with cochlear implants or hearing aids. These provide access to the speech signal and may facilitate the development of age-appropriate expressive and receptive language skills if accessed early, optimally before one year of age [13, 20]. The significant gross motor delay and balance issues caused by vestibular dysfunction in Type 1 can be supported with physical therapy (i.e., physiotherapy and occupational therapy). With no current regulatory-approved treatment for the vision impairment caused by Usher syndrome-associated retinitis pigmentosa, management largely involves the use of vision aids, adaptive technology and accessing supportive services. Overall, the multiple sensory impairments and the variation across disease subtypes results in complex individual management needs, requiring support from a wide variety of healthcare professionals.

Given their child’s complex management needs and the potential involvement of multiple professionals in their care, parents of children with Usher syndrome face a significant burden of decision-making and coordination of care. The coordination role can often include managing the diagnostic process, seeking out a team of healthcare professionals (including surgeons, speech pathologists, audiologists, ophthalmologists, optometrists, orthoptists, physiotherapists, and occupational therapists), scheduling and attending numerous appointments, implementing treatment plans, and seeking out funding to enable access to therapies. In addition, parents may need to take responsibility for communication between the healthcare professionals involved in their child’s care, as has often been reported by parents of children with rare diseases [15]. Parents of children with Usher syndrome have previously reported that these coordination tasks place a significant burden on them whilst they are trying to manage their own grief regarding the new diagnosis and contemplating their child’s future with deafblindness [29]. There is also substantial time pressure involved, as many of the common syndrome management strategies, such as cochlear implementation or vestibular rehabilitation therapy, are most effective when implemented in the first years of life [14, 30]. Parents anecdotally report feeling pressure to engage in early intervention strategies before their child turns five to maximise preparation for school, and to minimise the disruption to schooling from attending numerous appointments (personal communication, UsherKids Australia).

In addition to the challenges stemming from the syndromic features of the condition are the challenges relating to the low prevalence rates of Usher syndrome. Previous work has found that parents of children with rare diseases were largely dissatisfied with health professionals’ knowledge of their child’s condition and were often required to educate health professionals about the condition and appropriate management strategies [25]. They described a lack of disease-specific information, difficulties accessing support groups and challenges connecting with other parents of children with the same condition, all resulting in feelings of social isolation [25, 26]. At least one parent is often required to reduce or forego paid employment to allow the time and flexibility required to manage the child’s condition. This can contribute to financial and relational stress within the family unit [22]. These sentiments have been echoed by parents of children with Usher syndrome, who reported dissatisfaction with the level of knowledge of medical professionals and challenges in accessing support groups in their geographical location [29].

Whilst previous work, such as the survey study by Rabenn [29], has documented some of the psychosocial impacts experienced by parents of children with Usher syndrome, the support needs of parents have not been explored. Obtaining the views of parents may enable the identification of gaps in current support services, the development of evidence-based recommendations to organisations or support groups tasked with assisting this population, and the creation of effective tools or programs to target the support needs of this population. As discussed previously, Type 1 Usher syndrome is the most severe, due to both the range of symptoms and the earlier onset of sensory deficits. As such, the current study aims to explore the support needs of parents of children with Usher syndrome Type 1 when their child was aged 0 to 5 years.

## Methods

To address the study aim, semi structured interviews were conducted with Australian parents or carers of children with Type 1 Usher syndrome. Modified reflexive thematic analysis was used to analyse interview transcripts and identify themes across participants. The consolidated criteria for reporting qualitative studies (COREQ), a 32-item checklist, was employed to ensure comprehensive reporting of the current study’s methodology and findings [32]. This study was conducted in compliance with the tenets of the Declaration of Helsinki and was approved by the Human Research Ethics Committee of the University of Melbourne (22,754).

### Participants

Participants were recruited through the personal and professional networks of the authors. The advertising flyer with information about the study and methods for registering for study participation were circulated via social network postings and via emails to colleagues and to professional, clinical, and support organisations in the fields of vision, hearing, and genetics. In addition, the advertising flyer was attached to an electronic newsletter distributed to families of children with Usher syndrome, and to clinical and support organisations involved with these families. The newsletter shares information from an Australian Usher syndrome support group founded and managed by author ES (parent to a child with Usher syndrome and Master of Public Health student). Organisations were requested to advertise the study to potential participants in their network. Potential participants could access the Participant Information and Consent Form and register for the study through a link in the advertising flyer to a REDCap, a secure web application for electronic data capture hosted by the University of Melbourne, or by contacting the chief investigator (KG, audiologist and hearing researcher with expertise in cochlear implants).

Eligibility criteria were: (1) the parent or carer of a child who was diagnosed with Usher syndrome Type 1; (2) having been the child’s primary (or equal primary) caregiver (defined as the individual/s responsible for meeting the majority of the child’s needs) when the child was aged 0 to 5 years; (3) the child’s vision had deteriorated such that, in the opinion of the parent/carer, there was functional impact in everyday life; and (4) the child was currently aged between nine and 25 years. The minimum age of nine years of age was included so that the parent participants could retrospectively view their early experiences and support needs through the lens of their subsequent experience parenting a child with hearing, vision, and vestibular dysfunction. The maximum limit of 25 years (i.e., born in or following 1997) was set based on the accessibility of cochlear implantation in Australia for children as young as two years of age from 1997. The parents of children born prior to 1997 generally faced different treatment options and likely outcomes for their child. This study focused on the support needs of parents who had the opportunity to decide on cochlear implantation. If the participant was the parent/carer for more than one child who met the eligibility criteria, the participant was asked to focus on their support needs when the oldest of these children was aged zero to five years. Potential participants provided demographic information required to assess their eligibility for the study via REDCap.

### Materials

**Fig. 1:**
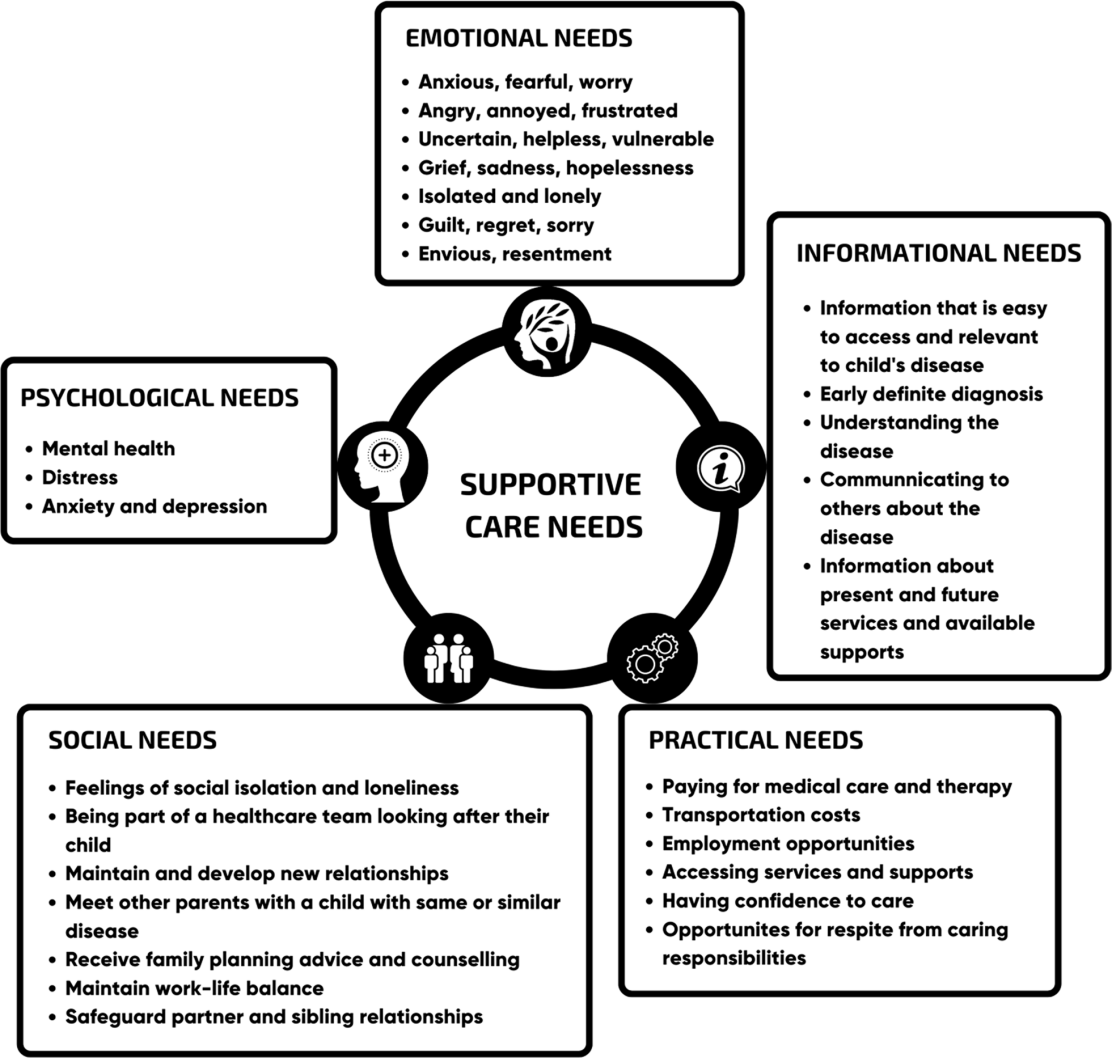
Parental supportive needs framework [27]

An interview guide developed by all authors consisted of open-ended questions intended to explore the participant’s support needs as the parent of a child with Usher syndrome when their child was aged zero to five years, with a particular focus on their support needs (refer to Supplemental Digital Content 1 for interview schedule). A parental supportive care needs framework developed by Pelentsov [27] was also used during the interview to prompt participants to identify their own support needs (see Fig. 1). This framework has been used extensively to explore parental support needs in the field of rare diseases [27]. The model consisted of five domains of support needs: emotional, informational, practical, social, and psychological. Whilst this framework was available to prompt parents to consider various supports they may have needed during their child’s early years, it was not prescriptive and parents were encouraged to discuss needs included and not included within the framework.

### Procedure

Written informed consent was captured using REDCap. Interviews were conducted by author FOH (MPhil (Med), B.Orth (Hons), clinician-researcher with expertise across both auditory and visual function) using the ZOOM videoconferencing software licensed to the University of Melbourne. FOH did not have a relationship with participants prior to interviewing, and upon interviewing each individual provided general information about her research experience and interest in the topic. No other individuals were present during interviewing. Audio-visual recordings of interviews were initially recorded directly to a University of Melbourne account in the secure AARnet CloudStor, and subsequently downloaded to a local computer for analysis. As such, no noting occurred during the interview.

At the start of the interview, the participant was shown the supportive needs framework as a prompt. The intention was to provide the participants with examples of support needs reported in previous research to assist them in recalling their own support needs. Participants were able to request to revisit the framework throughout the interview if they wished. The approximately 60-min interviews were conducted between March and June of 2022. After initial data analysis was complete, as described below, participants were recontacted via email and provided with preliminary outcomes (i.e., themes) for member checking purposes [5].

### Data analysis

Interview recordings were transcribed verbatim by authors FOH and LJ (project coordinator of Australian Usher syndrome support group and Doctor of Philosophy (Clinical Psychology) candidate). The transcriptions were reviewed and compared to the recordings several times to ensure accuracy. The transcriptions were then imported into NVivo 12 [28] for analysis. Braun and Clarke’s [7, 8] approach to reflexive thematic analysis was selected to guide the analysis as it is grounded within an interpretivist paradigm, which was congruent with the values of the research team and the aims of the study [12]. The analysis did, however, diverge from Braun and Clarke’s approach in several ways. Whilst an inductive approach was employed by allowing themes to emerge from the dataset, the use of the support framework to prompt the participants to consider various examples of support needs in a range of domains influenced both the data and analysis process. As is evident in the results section, overarching themes were organised in accordance with four of the five support need domains described by the framework [27], as these were consistent with data in the current study, whilst subthemes were developed inductively. A semantic approach to coding was utilised, as this was in line with the research team’s value of promoting participants’ voices whilst attempting to limit the interpretation applied to their identified needs.

To begin the data analysis, data familiarisation, initial code generation, and initial theme generation were conducted in a step-by-step manner [6, 9] by authors ES and LJ. Then, in a cyclical and iterative process, initial codes and themes were presented to all authors, who contributed to the reviewing, naming, and defining of themes, and identified additional codes and themes. Once all interview transcripts had been analysed in this manner, and no additional themes or refinements to existing themes were identified, the data analysis was complete. In the spirit of reflexive thematic analysis, all authors were included throughout the analysis in a collaborative manner to achieve richer interpretations of meaning, rather than to seek inter-rater reliability [11].

## Results

Participants were six mothers aged 46 to 59 years. No individuals dropped out of the study after expressing interest in participation. All participants had multiple children but only two participants had multiple children with Usher syndrome (P1 and P3 each had two children with Usher syndrome). Characteristics of the participants’ children with Usher syndrome (the first-born child with Usher syndrome for P1 and P3) are shown in Table 1.

**Table 1:** Participant-reported characteristics of their child with Usher syndrome

	Age of child (years)					
Activity/occurrence	P1	P2	P3	P4	P5	P6
Hearing loss diagnosis	0.7	Newborn	1	1	Newborn	0.7
Evidence of vestibular dysfunction	0.4	8	1	3	1	0.5
Usher syndrome diagnosis	5	10	11	15	3.5	14
Interview for current study	9	16	22	19	10	19

### Themes

Four main themes were identified as being central to the support needs of parents of children with Usher syndrome aged 0 to 5 years: (1) Social Needs, (2) Informational Needs (3) Practical Needs, and (4) Emotional Needs. Refer to Table 2 for the four main themes, their subthemes and example quotes.

**Table 2:** Main themes, and their sub-themes, identified as central to the support needs of parents of children with Usher syndrome aged 0 to 5 years

1. Social Needs	Example
a) Sharing lived experiences with other parents of children with Usher syndrome reduces isolation and loneliness	*“There's that intangible support of sitting with others that have walked in your shoes and it's that opportunity to talk with others that understand that you will not get anywhere else.”*
b) Support groups as facilitators for connection	*“I just think it's so invaluable. So, there is the shared information that is important that comes through that, parents often just say ‘I didn't know anything about that’. Everyone scribbling everything down, especially when it comes to NDIS … So, there's actually practical… information about the system and how to get what you need. You don't even know what you need until you hear someone else saying ‘this really worked for my child.”*
c) Pre-existing relationships did not provide sufficient support	*“I also remember thinking about my really close friendships … and none of them reached out, I was reaching out to them, saying, ‘I am going through the hardest thing I've ever gone through in my life, and I really need you here because this is beyond devastating, and overwhelming’ … I think they were all scared.”*
2. Informational Needs	
a) Accurate and timely information regarding diagnosis may relieve emotional distress	*“[We] would have been able to socialize with families in the same situation… [] would have been absolutely invaluable because that would have provided support."*
b) Parents needed to be provided with greater knowledge and information by professionals	*“I think you want to know what is Usher Syndrome? What are the different elements? What are the issues? What are the general expectations of Usher Syndrome? Certainly, that was something that we didn't get from the ophthalmologist or even genetics. Really the information [I have] was by doing my own research.”*
c) Parents required information to locate professionals with adequate knowledge and training	*"You spend a lot of time teaching the professionals that are engaged in the care of your child what the condition is and what the condition means, and that puts a lot of pressure on parents to be up to date with the information and managing the caseload like it is, like literally, a case worker job."*
d) Parents needed additional information on vestibular dysfunction	*“If there had been some better understanding of the processes that could assist [child’s] vestibular needs, then it would have taken a lot of time, effort, guesswork and money out of what we were trying to do for him.”*
e) Need for various sources of information	*“Links to websites and handouts [would have supported me in] a state of shock or anxiety”*
3. Practical Needs	
a) Respite allowed parents to recharge and better support their families	*"I was beyond exhausted and so that impacted everything…. I obviously had to work and so then just managing work with appointments. We're still getting the early intervention service, so there were those appointments. And all of that led to being beyond exhausted and then needing some psychological help. So the practical needs you often neglect because you're just running on empty and you don't know how to fix it. You are just too exhausted to try and fix it."*
b) Case coordination and collaborative care can remove burden from parents	*”It's just keeping the headspace to be on top of everything and knowing when you've got to go back for different appointments and what follow ups need to be done and just kind of being a caseworker for your child. So, if there's somebody else kind of doing that paperwork stuff and just making sure the appointments are happening and they're in the right order with the right people so you're not wasting time, sort of jumping from service to service, then that would be a really significant support for people.”*
c) Flexibility in employment options support parents in caring for their child/ren	*“I’m very lucky that I work for myself so that I’m able to [continue working]. I can tell you that probably from 0–5 (years), I wouldn't have been able to have a job. Because of the number of appointments and just trying to get her ready for school because she was diagnosed so late, and she wasn't implanted until she was two. There was so much catch up to do to make her ready to start school on time.”*
d) Financial support may relieve burdens associated with complex care needs	“*Parking vouchers, petrol vouchers or transport vouchers [would support] getting to numerous appointments… for each appointment you attend that's $30 to pay for parking and you're going to have to take at least half a day off work.”*
4. Emotional Needs	
a) Support required to cope with grief and loss	*“I wanted a counsellor who had experience with grief and loss associated with disability, who could understand … chronic grief and chronic loss. Someone who has enough experience with parents (of children) with disabilities that can … get to the crux of it, which is accepting an unexpected situation, accepting that it's really hard and everything that you're doing will probably be hard for a long time. That can reflect back and then really employ … psychological tools as needed for that person's level of grief, anxiety, depression, whatever it is…I would love to have met someone like that.”*
b) Confronting emotions regarding child’s progressive vision loss	*“Yeah, so I think understanding that the vision is going to be a long-term area of difficulty regarding that grief cycle. It's one that you're going to come back to…I think everything is fine but then their vision might drop off.”*

#### Theme 1: social needs

Parents detailed needing social support from various sources. They described substantial benefits to connecting with other parents of children with Usher syndrome and identified support groups to be useful in facilitating such connections. Parents also reported receiving inadequate support from their general support network.

### Sharing lived experiences with other parents of children with Usher syndrome reduces isolation and loneliness

The parents described their need to connect with other parents of children with Usher syndrome for support. When speaking about the support they received from other parents, one participant said, *‘the best gift you can have is that communication with other people in the same situation’.* Another described:

*“There's that intangible support of sitting with others that have walked in your shoes and it's that opportunity to talk with others that understand that you will not get anywhere else.”*

Parents identified other parents of children with Usher syndrome as being important sources of practical information, such as which services or funding they could access, recommendations regarding condition-informed healthcare professionals, and advice regarding early intervention:

*“Because you could just ask questions, when things come up, or ask ‘has anyone had experience with this?’ So, it's always helpful having that, and again across the lifespan with other families that have older adults with Usher’s that you can ask questions and get support from. That's helpful”.*

Gaining this practical information from other parents also had benefits in other areas as it led to an increased sense of connection, community, and support for the parents seeking the information.

*"The value of connecting with other families and other parents is essential from day dot, the support of talking with someone who's walked in your shoes. All of the informal information that you receive that the professionals don't necessarily know about or might not think to mention."*

In addition to general peer support, parents described wanting another parent of a child with Usher syndrome to be present whilst they received their child’s diagnosis, or to be available to meet with them shortly after. They believed that being able to hear about another family’s experiences would have relieved some of the distress that they experienced when they received their child’s Usher syndrome diagnosis.

*"You need to have either a person on board that's going through it, or whose children have it, so they can support you in the meeting and tell you it's actually not the end of the world. You will get through this."*

Parents reported a need to observe how older children with Usher syndrome were managing after receiving early intervention and after the onset of progressive vision loss. Peer support provided this opportunity, which then helped to alleviate the parent’s anxiety regarding their child’s future and allowed them to develop more realistic expectations for that future.

*“I think that's where parents and those social connections and seeing people living with Usher's comes in. It’s like well actually, it's not the end of the world. I just met a really happy, well-adjusted person who has a job, who happens to have Usher syndrome.”*

### Support groups as facilitators for connection

Parents needed support groups formed by, and for, parents of children with Usher syndrome. Those who had access to such support groups described them as a vital support. Some participants did not have access to support groups as there had yet to be an Usher syndrome-specific support group in Australia when their child was aged 0 to 5 years. These

participants reported that they would have benefitted from attending a group in their child’s early years. Support groups reduced the sense of isolation experienced by parents and increased their sense of connection to others undergoing similar experiences. Parents also reported support groups to be a helpful resource for gaining information on management strategies, early intervention services, and navigating disability funding:

*“I just think it's so invaluable. So, there is the shared information that is important that comes through...parents often just say ‘I didn't know anything about that’. Everyone scribbling everything down, especially when it comes to NDI*^]^^1^* … So, there's actually practical… information about the system and how to get what you need. You don't even know what you need until you hear someone else saying ‘this really worked for my child’”.*

### Pre-existing relationships did not provide sufficient support

In addition to the need for support from other parents of children with Usher syndrome was the need for social support from friends, family, and colleagues. Parents reported feeling that they did not receive sufficient support from their network of close contacts, particularly in the early stages following their child’s diagnosis:

*“I also remember thinking about my really close friendships … and none of them reached out, I was reaching out to them, saying, ‘I am going through the hardest thing I've ever gone through in my life, and I really need you here because this is beyond devastating, and overwhelming’ … I think they were all scared.”*

Parents reported a desire to have a resource that they could provide to family, friends, and colleagues describing what Usher syndrome is and the ways in which support could be provided to the affected child and their family. Parents felt this would reduce the burden on

themselves to educate others about the syndrome and to reduce the uncertainty others may experience when considering how to best support the family.

#### Theme 2: informational needs

Parents described needing support through information, which was often unmet. These needs related to being provided with an accurate and timely diagnosis, receiving detailed information from treating professionals, locating professionals with appropriate knowledge and training, accessing specific information regarding vestibular dysfunction, and needing various sources of information.

### Accurate and timely information regarding diagnosis may relieve emotional distress

Parents reported finding the diagnostic process to be challenging and emotional. They described feeling that they had been provided with inaccurate information as they initially only received a diagnosis of hearing impairment for their child, only to receive a diagnosis of Usher syndrome, sometimes years later. Parents were required to advocate for their child by pressuring healthcare professionals to complete further diagnostic testing. This process often involved consulting several professionals before the Usher syndrome diagnosis was made. Parents felt genetic testing needed to be immediately available for children who returned an abnormal result from screening of newborn hearing. They believed that this would reduce the emotional impact on the parents of receiving an Usher syndrome diagnosis months or years after coming to accept their child’s hearing impairment. They also felt that this would support parents in making informed treatment and management decisions. Early diagnosis was also identified as a mechanism for supporting parents’ needs in other areas, as they *“would have been able to socialize with families in the same situation”,* which* “would have been absolutely invaluable because that would have provided support."*

### Parents needed to be provided with greater knowledge and information by professionals

Parents reported needing more support in accessing information relating to Usher syndrome. Parents consistently described feeling that, once their child was diagnosed with Usher syndrome, their need for information about the syndrome was not met. Several parents reported that the professional providing the diagnosis did not provide them with any additional information beyond the diagnosis itself.

*“I think you want to know what is Usher Syndrome? What are the different elements? What are the issues? What are the general expectations of Usher Syndrome? Certainly, that was something that we didn't get from the ophthalmologist or even genetics. Really the information [I have] was by doing my own research.”*

Following the diagnostic appointment, another parent recalled having *"no clue what was going on. It was just like 'Here is the diagnosis'. I had no clue that there were three types of Usher syndrome. I had zero information.”*

As they were not provided with adequate information from professionals, parents commonly sought information about Usher syndrome from the internet. Parents described a preference for being provided with an information pack by the healthcare service in the early period following diagnosis. They felt this information was necessary for them to understand their child’s experience, the possible treatment and management options, and the potential trajectory of the syndrome.

### Parents required information to locate professionals with adequate knowledge and training

Parents reported needing information on healthcare professionals who had adequate knowledge of Usher syndrome and management strategies. Parents described professionals they consulted as lacking knowledge in identifying signs of Usher syndrome, as well as an understanding of the symptoms and progression of the condition. They also reported that professionals often did not provide syndrome-specific treatment and management. This perceived lack of professional knowledge resulted in parents needing to seek information themselves as they felt they could not rely on professionals to provide such information:

*"You spend a lot of time teaching the professionals that are engaged in the care of your child what the condition is and what the condition means, and that puts a lot of pressure on parents to be up to date with the information and managing the caseload like it is literally a case worker job."*

Additionally, parents needed healthcare professionals to provide empathetic care which was specific to the additional burden of a rare disease. Parents illustrated this need by describing the harmful comments made by professionals, such as ‘*your kid’s got Ushers [there’s] no hope’.* Parents also referred to their need to engage with professionals to discuss the psychological impacts of the diagnosis but being unable to locate specialised professionals who worked with the ambiguous grief and loss associated with having a child with a disability.

Following diagnosis, many parents described being unsure of which healthcare providers they needed to consult and how to access professional services such as early intervention. Parents reported needing support in finding and accessing such services:

*“I had to hunt down these people …I had to do the research myself. It wasn't like there was a directory! No there wasn't [anyone saying] they are going to help you.”*

Parents reported a need for professionals who had specialised knowledge of Usher syndrome in their field of expertise. They expressed a need for a ‘*list of clinicians that you can go to that have expertise in this area’.* This was particularly relevant in the management of vestibular dysfunction, as parents noted a lack of physiotherapists with expertise in this area or difficulties in locating the appropriate professional. The challenge of accessing appropriate professional services was further compounded for families who lived outside of major cities, as they often had to travel long distances to access services:

*“This had an impact on my emotional needs and psychological needs. I draw a direct correlation there because of the travel involved. We were expected to be at the implant clinic every week, and I did that for four years.”*

### Parents needed additional information on vestibular dysfunction

Parents identified a particular need for information regarding the vestibular dysfunction component of Usher syndrome, as such information was hard to find. Parents reported there being *‘no information about the vestibular [dysfunction] … and there still are very few people that understand the vestibular dysfunction and how to manage it’.* Another parent stated that: *“If there had been some better understanding of the processes that could assist [child’s] vestibular needs, then it would have taken a lot of time, effort, guesswork and money out of what we were trying to do for him”.*

### Need for various sources of information

Parents reported that they needed information that was in various formats and delivered in different styles, so that they, as an individual, could access the information they required. This included flexibility in the location of appointments or support meetings, so that they could be accessed online if preferred. One parent needed ‘*links to websites and handouts’* as they had felt unable to process verbal information following their child’s diagnosis due to being in *‘a state of shock or anxiety’*.

#### Theme 3: practical needs

Parents described a number of practical supports they required to manage day-to-day responsibilities and costs, including support so that they could take time for their own self-care, flexibility in work hours to attend appointments, and financial support.

### Respite allowed parents to recharge and better support their families

Parents needed respite care so that they could take time to care for themself in order to be able to care for their child and family. Parents reported feeling overwhelmed by the tasks they needed to do, such as attending appointments and acting as a case coordinator for their child, which resulted in psychological and physical fatigue:

*"I was beyond exhausted and so that impacted everything.... I obviously had to work and so then just managing work with appointments. We're still getting the early intervention service, so there were those appointments. And all of that led to being beyond exhausted and then needing some psychological help. So, the practical needs you often neglect because you're just running on empty and you don't know how to fix it. You are just too exhausted to try and fix it."*

They described needing opportunities to engage in self-care so they could better support their family members. Respite also allowed parents to “*have the freedom to be able to catch up with people and have those conversations about how you're really feeling. I think if people have the ability to have that in place it can then support their emotional needs as well.”*

### Case coordination and collaborative care can remove burden from parents

Parents reported a need for collaborative care from their healthcare team. Some considered that this may be best achieved via a case coordinator with responsibility for facilitating communicating amongst the team and between the team and the family, and for organising additional supports based on the family’s needs.

*"They all do their own thing. So, the hearing people are over there, the visual people are over there, the vestibular physio is over there and none of them really talked to each other very much. They focus on their one special area. The NDIS, the system in Australia, doesn't understand the integrated nature of these disabilities, when they come in the form of a syndrome.”*

Parents described having to take on a case manager role themselves and detailed the benefits they believe would occur as a result of having an external case manager:

*”It's just keeping the headspace to be on top of everything and knowing when you've got to go back for different appointments and what follow ups need to be done and just kind of being a caseworker for your child. So, if there's somebody else kind of doing that paperwork stuff and just making sure the appointments are happening and they're in the right order with the right people so you're not wasting time, sort of jumping from service to service, then that would be a really significant support for people.”*

This concept of a case manager was highlighted to be of particular importance to parents in the early stages following diagnosis:

*” I guess it would have been nice to have an initial case manager, someone who can just go ‘Okay, what do you need?’, ‘How can we link you in?’, ‘What information do you need?’… There's this, there's that, and just someone to help you along on that journey a little bit.”*

### Flexibility in employment options support parents in caring for their child/ren

Parents needed support from their employer, or to be self-employed, to work flexibly and allow time to attend appointments in their child’s early years, in order to continue working:

*“I’m very lucky that I work for myself so that I’m able to [continue working]. I can tell you that probably from 0-5 (years), I wouldn't have been able to have a job. Because of the number of appointments and just trying to get her ready for school because she was diagnosed so late, and she wasn't implanted until she was two. There was so much catch up to do to make her ready to start school on time.”*

Other parents reported needing to forego paid employment so they could attend all necessary appointments and care for their child.

### Financial support may relieve burdens associated with complex care needs

Parents needed financial support for the significant costs associated with having to attend numerous appointments, including the cost of the appointment, travel and parking, and foregoing paid work to attend appointments. Parents suggested that the implementation of vouchers for parking or transportation would have alleviated some of the financial burden they experienced.

“*Parking vouchers, petrol vouchers or transport vouchers [would support] getting to numerous appointments… for each appointment you attend that's $30 to pay for parking and you're going to have to take at least half a day off work.”*

#### Theme 4: emotional needs

All parents described needing some form of emotional support. Parents needed emotional support for themselves so that they could then provide positive support to their child. They described particularly needing emotional support regarding their experience of grief and loss as well as their concerns regarding their child’s future. For some, this support was sought from their family and friends, whereas others sought support from a psychologist.

### Support required to cope with grief and loss

Parents highlighted the need for support in managing their feelings of grief regarding the experiences or opportunities their child may miss out on as a result of having Usher syndrome:

*“It was just so much for me to bear, and I just thought it was the end of the world, like they're never going to get a job. They're never going to get married."*

They described feeling ‘stuck in the grief cycle and unable to move on’. Some found relief in talking to their family and friends, whilst others described needing additional support through formal mental health supports. However, some parents were unable to find a professional with expertise in working with the unique challenges and grief associated with having a child with a disability:

*“I wanted a counsellor who had experience with grief and loss associated with disability, who could understand … chronic grief and chronic loss. Someone who has enough experience with parents [of children] with disabilities that can … get to the crux of it, which is accepting an unexpected situation, accepting that it's really hard and everything that you're doing will probably be hard for a long time. That can reflect back and then really employ … psychological tools as needed for that person's level of grief, anxiety, depression, whatever it is…I would love to have met someone like that.”*

### Confronting emotions regarding child’s progressive vision loss

Parents described needing emotional support in preparing for their child’s vision loss. This need was greater due to the lack of vision-related treatment strategies:

*“With the vision, it's like there's nothing I can do, it’s going to get worse and that’s hard.”*

Additionally, the progressive nature of vision loss results in parents re-experiencing acute grief at various times:

*“Yeah, so I think understanding that the vision is going to be a long-term area of difficulty regarding that grief cycle. It's one that you're going to come back to…I think everything is fine but then their vision might drop off.”*

Although they needed vision-related services, parents described engaging with such services to be confronting for both themselves and their child, which sometimes meant they would avoid engagement:

*“I was too afraid to access vision services when he was young because I felt they were very confronting because there was no division between those with total vision loss and a kind of ongoing vision loss, so I think that still needs to be addressed.”*

Parents described challenges related to identifying appropriate parenting strategies, particularly in regard to deciding how to have discussions with their child regarding their vision loss.

They felt supported by other parents with lived experience and formal psychology services in navigating these challenges.

## Discussion

This study explored the support needs of parents of children with Usher syndrome Type 1 when their child was aged between 0 and 5 years. Six mothers reported extensive support needs, in a variety of areas, across the first five years of their child’s life. These needs related to areas of social, informational, practical, and emotional support.

Parents described a need for support that was often unmet. They reported needing to connect with and obtain support from peers and others in the Usher syndrome community in an individual and group setting, as well as via social media. An Australian support group for families of children with Usher syndrome did not exist for these participants in the first years of their child’s life, making it challenging to connect with parents of children with this rare disease. Access to disease-specific support groups is a commonly described challenge in the rare disease literature [25, 31]. Up to 60% of parents of children with a rare disease have described being unable to access an Australian disease-specific support group [1], despite such groups being an important protective factor (von [35]). The lack of an appropriate peer support group impedes parents' access to crucial peer support, which participants in the present study identified as an important source of practical information, as well as reassurance regarding their child’s future happiness and quality of life.

Parents also reported a need for information throughout the diagnostic and later management stages. This included needing information about the most appropriate clinical management pathway, support to find and access the required health professionals, and accurate, up-to-date information from their treating health professionals. The parents reported that these needs were not adequately met. These findings are consistent with reports from the rare disease literature, with parents often being more knowledgeable about their child’s disease than the treating professionals [15], and clinicians often lacking formal education and training regarding rare disease management [21]. Furthermore, a recent survey of key allied health professionals (optometrists, orthoptists, and audiologists) involved in Usher syndrome care found that these professionals had significant knowledge gaps regarding the characteristics of Usher syndrome and recommended management strategies [3].

Parents required the practical supports of respite, flexible employment, and financial assistance. Respite was reported to allow parents to engage in their own self-care, the importance of which is, again, supported by the broader rare disease literature [26]. Despite its value, it is challenging for parents of children with a rare disease to access respite, with up to 77% of parents in Australia struggling to find a respite-carer for their child [1]. The existing literature also supports the current finding that parents of children with rare and/or complex conditions require flexible employment in order to be able to provide optimal care to their child [4]. Without such flexibility, many parents, most often mothers, ultimately forego paid employment [4], resulting in financial and psychosocial consequences for the individual parent and family.

All parents emphasised the importance of emotional support, particularly to assist them in processing their grief and loss, as well as their concerns regarding their child’s future. Whilst this emotional support was provided by friends and family in some cases, most parents reported needing to access formal psychological support. This need is consistently reported in the literature, which has identified parents of children with rare diseases as experiencing higher rates of anxiety, depression, and insomnia, as well as poorer quality of life, in comparison to controls [24]. In spite of this clear need, many of our participants, as well as participants from previous Australian research [1, 27], reported difficulties in locating mental health professionals with expertise in the emotional needs of parents of children with a rare disease. From both our findings and those from prior research, it is unclear whether this difficulty is due to a fundamental lack of professionals with these specific skills, or if it is due to a lack of clear pathways to identifying appropriate experts. It may be that mental health professionals need upskilling in providing support to parents of children with a rare disease. Alternatively, a clear and easy to use resource to support families in locating those with expertise in this area may effectively address this need.

### Implications

The findings of this study highlight the need for medical and allied health professionals in relevant fields to have improved knowledge of Usher syndrome, including its onset, symptoms, progression, long-term outcomes, and treatment options. As the average age of diagnosis continues to decrease [10], greater opportunities are being created to provide early intervention to children, such as vestibular rehabilitation therapy or orientation and mobility training prior to vision loss. Without up-to-date knowledge, health professionals are unlikely to understand the critical value of such early intervention or to be aware of service providers to which they can refer parents. Optimally, best practice guidelines would be developed to support health professionals to provide evidence-based and consistent care to children with Usher syndrome, including referrals to other health professionals and service providers. Medical and allied health professional bodies have a role to play in providing professional development, education materials, and resources on rare conditions such as Usher syndrome to upskill their members. Ultimately, such bodies, and individual members with a particular interest, must develop the much-needed best-practice guidelines.

The results of the current study also highlight several systemic changes that are needed to support families in the broader rare disease community. Although they have a pivotal role in supporting and connecting children, parents, and families, disease-specific support groups are rarely integrated into the healthcare system. This impacts their visibility to medical and health professionals and consequently limits referrals to such groups. Incorporating support groups within the healthcare system may improve accessibility. This may also assist in the financial challenges many support groups face, as there are currently no clear funding pathways for the establishment or maintenance of groups [16]. These financial challenges currently obstruct the establishment of much needed groups, as well as the capacity of such groups to provide high quality and consistent support to families [21]. The present results also highlight the need for case coordinators who would substantially reduce the burden placed on parents of children with Usher syndrome. The value of case coordination has been empirically supported by past research [36], yet the path to implementation as a standard of care is currently unclear.

### Limitations

The homogenous nature of the study sample impacts the generalisability of findings in several ways. Firstly, despite attempting to recruit participants from a variety of channels, respondents were from one channel only, namely one support organisation. This may have resulted in an overemphasis in the results on the role or benefits of such groups, possibly limiting the generalisability of findings to those less connected to support services. This is a common issue described in the support group and rare disease literature (e.g., [4]) and additional strategies for recruiting participants from a wider variety of sources should be considered in future research. Secondly, the sample was comprised exclusively of mothers, as the families who volunteered to participate all identified the mothers to be the primary caregiver for their children. Future research is needed to explore the support needs of children diagnosed with Usher syndrome, as well as their fathers, siblings, and other carers. Finally, the participants all resided in Australia, and this may limit the generalisability of findings to parents internationally, although the focus of the interviews was on support needs, not on how local services met those needs.

## Conclusion

Parents of children with Usher syndrome Type 1 interviewed in this study reported extensive support needs across a variety of areas: peers, support group organisations, and formal services. Parents reported both psychological, practical, and informational needs. Some support needs were immediate, such as peer-support during the diagnostic process, whilst others related to the potential trajectory of their child’s condition, such as reassurance regarding their child’s future quality-of-life. This research provides valuable information for support groups, policy makers, individual healthcare professionals, and professional governing bodies to use in educating stakeholders and in developing and implementing best-practice guidelines, particularly around early intervention and support.

## Data Availability

The materials and deidentified data from the current study are available from the corresponding author on reasonable request. Data has not been made publicly available to protect participant’s privacy. https://www.springernature.com/gp/authors/research-data-policy/data-availability-statements

## Footnotes

1 an Australian Federal Government initiative designed to fund ‘reasonable and necessary’ costs, such as therapies, support workers and assistive technology, associated with ‘permanent and significant disability’ [2].
