# Correction to: Exploring the support needs of Australian parents of young children with Usher syndrome: a qualitative thematic analysis

**DOI:** 10.1186/s13023-024-03184-z

**Published:** 2024-04-18

**Authors:** L. Johansen, F. O’Hare, E. R. Shepard, L. N. Ayton, L. J. Pelentsov, L. S. Kearns, K. L. Galvin

**Affiliations:** 1 UsherKids Australia, Mordialloc, VIC, Australia; 2 Department of Optometry and Vision Sciences, The University of Melbourne, Parkville, VIC, Australia; 3 Department of Surgery (Ophthalmology), The University of Melbourne, Parkville, VIC, Australia; 4 Centre for Eye Research Australia, East Melbourne, VIC, Australia; 5 Clinical and Health Sciences, University of South Australia, Adelaide, SA, Australia; 6 Department of Audiology and Speech Pathology, Faculty of Medicine, Dentistry and Health Sciences, The University of Melbourne, Parkville, SA, Australia; 7 Royal Victorian Eye and Ear Hospital, East Melbourne, SA, Australia

**Correction: Orphanet Journal of Rare Diseases (2024) 19:129**

10.1186/s13023-024-03125-w

Following publication of the original article [1], we have been notified that one of the auhtors’ last name was published incorrectly.

It is now as follows:

L. J. Pelenstov 5

It should be as follows:

L. J. Pelentsov 5

The original article was updated.
